# Supplementary material for: Spatial Distribution, Antioxidant Capacity, and Spore Germination-Promoting Effect of Bibenzyls from Marchantia polymorpha
Source: Antioxidants (Basel). 2022 Oct 31;11(11):2157. doi: 10.3390/antiox11112157 (PMC9686712; doi:10.3390/antiox11112157)
Supplement: Supplementary file 1 [file antioxidants-11-02157-s001.zip › Supporting Information.pdf]

Supporting Information

Article

Spatial Distribution, Antioxidant Capacity, and Spore Germination−Promoting Effect of Bibenzyls from *Marchantia polymorpha*

Jiao-Zhen Zhang †, Chan Wang †, Ting-Ting Zhu, Jie Fu, Hui Tan, Cheng-Min Zhang, Ai-Xia Cheng * and Hong-Xiang Lou *

Key Laboratory of Chemical Biology of Natural Products (Ministry of Education), School of Pharmaceutical Sciences, College of Medicine, Shandong University, Jinan 250012, China

† These authors contributed equally to the work.

* Correspondence: aixiacheng@sdu.edu.cn (A.-X.C.); louhongxiang@sdu.edu.cn (H.-X.L.)

Table S1. Primers used for RT-PCR in this study.

**Primers**	**Primer Sequence(5'-3')**
Mpactin-RTF	GGCTGTTTTGTCGCTGTACG
Mpactin-RTR	CGCAGTGGTGGTGAAAGAGT
MpSTCS1A-RTF	TTCGGTGATGGAGCTTCAGT
MpSTCS1A-RTR	TGGAAATCAGACCGGGAACA
Mp4CL-RTF	CACCCATCATTGTCGCCTTG
Mp4CL-RTR	ACGAGGAGGGAGGGATTTAC

Table S2. Identification results of archegoniophore from female M. polymorpha by Compound Discoverer software (Attached)

Table S3. The identification result of 8 reference standards.

NO.	Rt（min）	$[M-H]^-$ (*m/z*)	Molecular formula	Identification
**1**	11.43	257.08193	$C_{15}H_{14}O_4$	Lunularic acid (LA)
**2**	13.23	455.15001	$C_{28}H_{24}O_6$	Marchantin B (MB)
**3**	14.19	423.16018	$C_{28}H_{24}O_4$	Isoriccardin C (IRC)
**4**	14.51	439.15510	$C_{28}H_{24}O_5$	Marchantin A (MA)
**5**	14.87	423.16018	$C_{28}H_{24}O_4$	Isorricardin D (IRD)
**6**	15.67	423.16018	$C_{28}H_{24}O_4$	Marchantin C (MC)
**7**	16.02	423.16018	$C_{29}H_{24}O_4$	Isomarchantin C (IMC)
**8**	16.58	423.16018	$C_{28}H_{24}O_4$	Neomarchantin A (NMA)

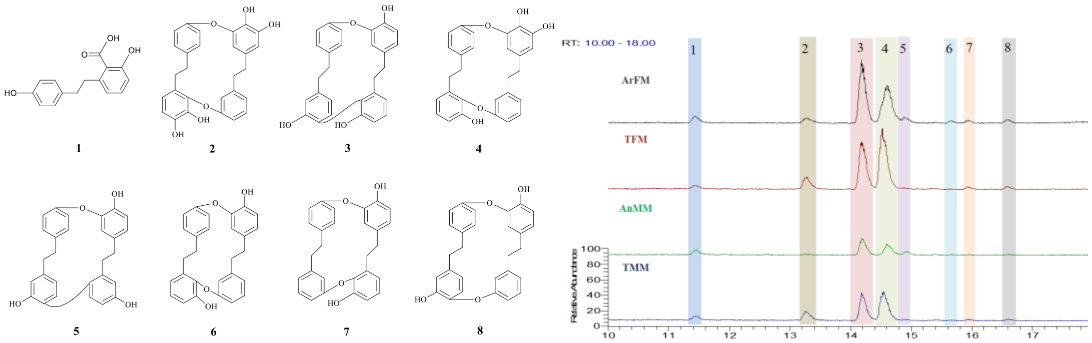

Figure S1. Structures of the 8 reference standards and total ion current (TIC) chromatograms of four plant parts from female and male *M. polymorpha* (ArFM, TFM, AnMM and TMM) in the negative-ion mode.

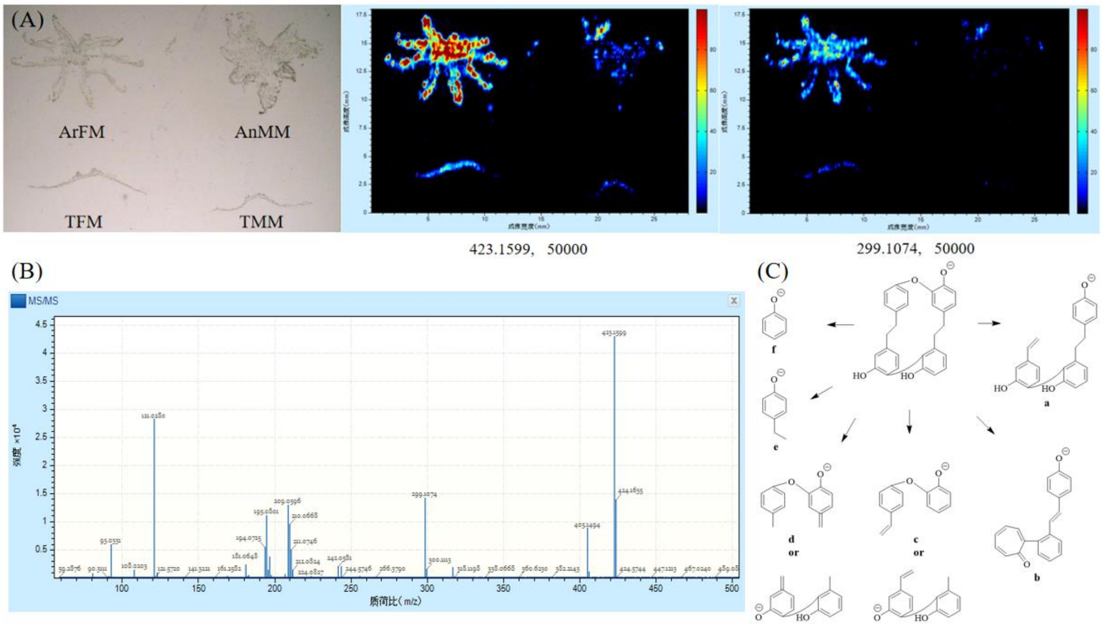

Figure S2 (A) Optical images of cross sections from four parts (ArFM, TFM, AnMM and TMM) and AFADESI images of parent ion 423.1599 and characteristic fragment ion 299.1074. The intensity thresholds are both 50000. (B) MS/MS image of the parent ion 423.1599. (C) Common cleavage mode of Isoriccardin C.
